# Primary murine mucosal response during cephalosporin‐induced intestinal colonization by *Enterococcus faecium*

**DOI:** 10.1002/mbo3.602

**Published:** 2018-02-27

**Authors:** Antoni P. A. Hendrickx, Denise van de Kamer, Rob J. L. Willems

**Affiliations:** ^1^ Department of Medical Microbiology University Medical Center Utrecht Utrecht The Netherlands

**Keywords:** antibiotic‐resistant *E. faecium*, cadherin‐17, CD11b^+^IgA^+^ cells, E‐cadherin, intestinal colonization

## Abstract

Hospitalized patients are often administered antibiotics that perturb the gastrointestinal commensal microbiota, leading to outgrowth of antibiotic‐resistant bacteria, like multidrug‐resistant *Enterococcus faecium*, subsequent spread, and eventually infections. However, the events that occur at the initial stage of intestinal colonization and outgrowth by multidrug‐resistant *E. faecium* within the antibiotic‐treated host have not been thoroughly studied. Here, we describe and visualize that only 6 hr after cephalosporin treatment of mice, the Muc‐2 mucus layer is reduced and E‐cadherin junctions were altered. In contrast, the cadherin‐17 junctions were unaffected in antibiotic treated mice during *E. faecium* colonization or in untreated animals. *E. faecium* was capable to colonize the mouse colon already within 6 hr after inoculation, and agglutinated at the apical side of the intestinal epithelium. During the primary stage of gastrointestinal colonization the number of IgA^+^ cells and CD11b^+^IgA^+^ cells increased in the lamina propria of the colon and mediated an elevated IgA response upon *E. faecium* colonization.

## 1 INTRODUCTION

In healthy humans the microbiota remains separated from the intestinal lamina propria and underlying tissue by mucosal barrier defenses to protect the host from severe inflammatory responses (Buffie & Pamer, 2013; Chu & Mazmanian, 2013; Round & Mazmanian, 2009). The most important defense mechanisms include a mucus layer for the physical segregation of microbes from the epithelium, a monolayer of intestinal epithelial cells secured by cell‐cell junctions, and secretion of immunoglobulin A (sIgA) (Gallo & Hooper, 2012; Kaetzel, 2014; Macpherson, McCoy, Johansen, & Brandtzaeg, 2008; Mathias, Pais, Favre, Benyacoub, & Corthésy, 2014; Turner, 2009). The intestinal mucus layer consists of mucin‐2 (Muc‐2), a glycoprotein produced by Goblet cells (Johansson, Larsson, & Hansson, 2011; Johansson, Sjövall, & Hansson, 2013). In the colon, Muc‐2 establishes a net‐like layer of which the inner mucus layer is impenetrable to bacteria thereby protecting the epithelium (Johansson et al., 2008). Intestinal epithelial cells are connected by various cell‐cell adhesion junctions, including the E‐cadherin adherens junction (Brieher & Yap, 2013; Ivanov & Naydenov, 2013; Mehta, Nijhuis, Kumagai, Lindsay, & Silver, 2014; Rodriguez‐Boulan & Macara, 2014). Cadherin‐17, also known as liver‐intestine cadherin, is not found in the E‐cadherin junction, but can be distributed along the basal, lateral, and apical plasma membranes to mediate cell‐cell adhesion (Angres, Kim, Jung, Gessner, & Tauber, 2001; Baumgartner, 2013; Berndorff et al., 1994; Dantzig et al., 1994; Wendeler, Drenckhahn, Gessner, & Baumgartner, 2007).

Below the epithelial monolayer, IgA^+^ intestinal plasma cells secrete sIgA antibody (Brandtzaeg, 1973, 2013; Mathias et al., 2014). Following secretion into the lumen, sIgA shapes bacterial communities and protects the host from the microbiota by immune exclusion (Fagarasan, 2006; Macpherson & Slack, 2007; Macpherson et al., 2008; Slack et al., 2009). Intestinal IgA^+^ plasma cells in mice could be divided into two populations based on surface integrin CD11b expression (Kunisawa et al., 2013). The CD11b^+^IgA^+^ plasma cells proliferate vigorously, produce more sIgA than CD11b^‐^IgA^+^ cells, require microbial stimulation, and contribute to initial intestinal IgA responses (Kunisawa et al., 2013).

During antibiotic treatment, the intestinal mucosal barrier defenses are greatly challenged. Antibiotic administration deregulates gut homeostasis (Brandl et al., 2008; Vaishnava et al., 2011), leads to a reduced Muc‐2 mucus layer and affects E‐cadherin junctions (Hendrickx et al., 2015; Wlodarska et al., 2011), and mediates outgrowth of antibiotic‐resistant pathogens (Becattini, Taur, & Pamer, 2016; Caballero & Pamer, 2015; Hendrickx et al., 2015; Ubeda et al., 2010). Similarly, in immune compromised patients receiving antibiotics, multiantibiotic‐resistant *Enterococcus faecium* becomes the dominant intestinal species (Ruiz‐Garbajosa et al., 2012; Ubeda et al., 2010; Van Harten, Willems, Martin, & Hendrickx, 2017). Enterococcal infections can subsequently occur through translocation from the intestine into the bloodstream or via fecal contamination (Arias & Murray, 2012).

However, the initial events and factors at play during antibiotic treatment and *E. faecium* colonic outgrowth are not understood. Therefore, the major goal of this study was to investigate the early primary barrier defenses (mucus layer, cell‐cell junctions, plasma cell response) in the colons of mice during cephalosporin treatment and multiantibiotic‐resistant *E. faecium* outgrowth in a mouse gut colonization model.

## 2 MATERIALS AND METHODS

### 2.1 Bacterial strain

*E. faecium* strain E1162 (Hendrickx et al., 2015; Paganelli et al., 2017) was grown aerobically overnight on Trypticase soy agar II supplemented with 5% sheep blood (TSA‐SB) at 37°. The MIC of *E. fecium* strain E1162 for ceftriaxone is >512 μg/ml and for cefoxitin 256 μg/ml.

### 2.2 Mice

Twenty‐four 10‐week‐old specific pathogen‐free wild‐type Balb/c mice were purchased (Charles River Laboratories, USA) and housed in the animal facility department of the Academic Medical Center Amsterdam, The Netherlands. The rooms had controlled temperature and a 12‐hr light‐dark cycle and the animals were acclimated for one week prior to the experiment. The mice received standard rodent chow and water ad libitum.

### 2.3 Ethics statement

The Animal Care and Use Committee (DEC) of the University of Amsterdam, The Netherlands reviewed and approved the mouse intestinal colonization experiment (number DIX74). The experiment was conducted following the Dutch law on ‘Experiments on Animals’ (Wet op de Dierproeven, WOD), in which the European Union guideline 2010/63/EU is implemented per 18/12/2014.

### 2.4 Mouse gastrointestinal colonization experiment

**Figure 1:**
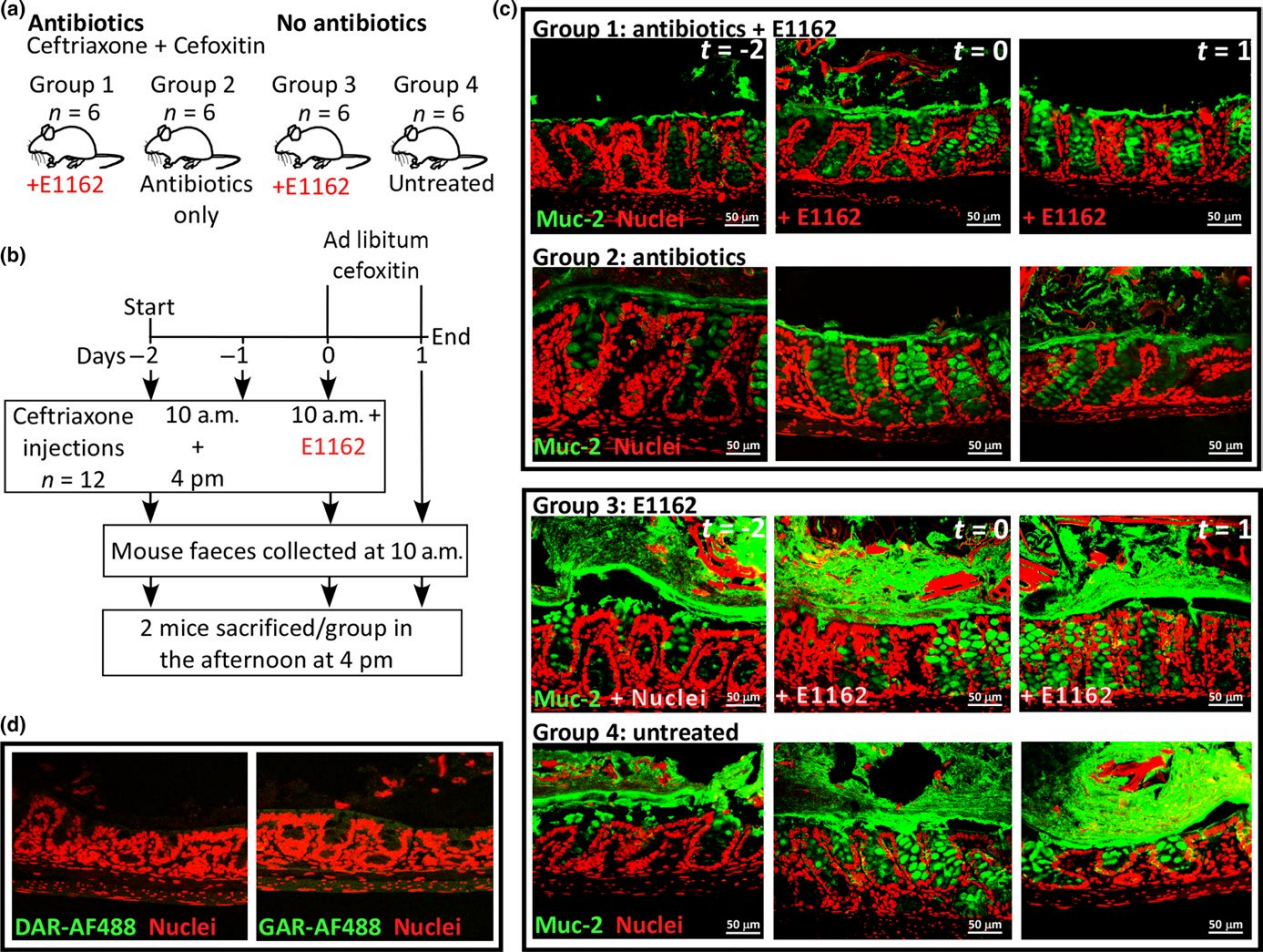
Set‐up of the *E. faecium* mouse intestinal colonization experiment and the mucus layer. (a) Group 1: cephalosporin antibiotic treated mice inoculated with *E. faecium* E1162 (n* *=* *6). Group 2: mice treated with antibiotics only (n* *=* *6). Group 3: untreated mice inoculated with *E. faecium* E1162 (n* *=* *6). Group 4: untreated mice (n* *=* *6). (b) Mice received ceftriaxone injections twice a day, at 10 am and 4 pm, on days −2, and −1 (n* *=* *12) and one ceftriaxone injection on 10 am on day 0, and were left on drinking water supplemented with cefoxitin from day 0. On day 0, the mice were orally inoculated with *E. faecium* E1162 (n* *=* *12). Feces was sampled at 10 am in the morning. Two mice per group were sacrificed in the afternoon at 4 pm on days −2, 0, and 1. (c) The mucus layer was stained in antibiotic treated mice that were inoculated with *E. faecium* E1162, mice treated with antibiotics only, and untreated mice inoculated with E1162 and untreated mice on days −2, 0, and 1. In green: rabbit anti‐mouse Muc‐2 +  goat anti‐rabbit Alexa Fluor 488. In red: TO‐PRO‐3 nucleic acid (nucleus) stain. (d) Conjugate controls used in this study; DAR‐AF488 = Donkey anti‐Rabbit Alexa Fluor 488 and GAR‐AF488 = Goat anti‐Rabbit Alexa fluor 488

Twelve mice were injected subcutaneously on day −2, −1, and 0 with ceftriaxone (Roche, The Netherlands; 100 μl per injection, 12 mg/ml; 5 injections in total) 2 days prior to inoculation of *E. faecium* E1162 (Figure 1a). Mice received an injection two times a day (early in the morning at 10 am, and late in the afternoon at 4 pm) and early in the morning on the day of inoculation (day 0) and they were left on ad libitum cefoxitin antibiotics (0,125 g/L) in their sterile drinking water. In addition, 12 mice received 0.9% NaCl injections, and were left on sterile drinking water without antibiotics. More specifically, six mice (group 1) were treated with ceftriaxone and cefoxitin and were inoculated with 2x10^9^ CFU *E. faecium* E1162 on day 0. As a control, six mice were treated with ceftriaxone and cefoxitin alone as described above, but were not inoculated with *E. faecium* (group 2). Six mice (group 3) received 0.9% NaCl injections and were inoculated with 2 × 10^9^ CFU *E. faecium* (E1162) on day 0. As another control, six mice were left untreated (group 4). Two mice of each group were anesthetized with ketamine (75 mg/kg) and medetomidine (1 mg/kg) and sacrificed by cervical dislocation on each day −2, 0, and 1 in the afternoon. Sacrificing of mice on day −2 was done 6 hr after the first ceftriaxone injection (4 pm), on day 0, 6 hr after *E. faecium* E1162 inoculation (4 pm), and on day 1 also at 4 pm. Colon was removed during necropsy and fixed in Carnoy's fixative for histopathology and in 2% glutaraldehyde for scanning electron microscopy. Mouse fecal pellets (2–3 fecal pellets/ml 0.9% NaCl) were collected on day −2, −1, 0, and 1 and homogenized in 1 ml 0.9% NaCl. It is important to note that the collection of feces at day −2 and −1 was done before the ceftriaxone injections (groups 1 and 2) and at day 0 before the inoculation with *E. faecium* E1162 (groups 1 and 3). The fecal extracts were spun down at 20,000 x g for 10 min. The supernatant was transferred into a new tube spun down and the cleared fecal extracts were stored at −20°C.

### 2.5 Antibodies

Polyclonal Rabbit anti‐mouse IgG antibody directed to CD11b was purchased from Abcam (The Netherlands). Goat anti‐mouse IgG to mouse E‐cadherin, Rabbit anti‐mouse to mouse cadherin‐17 were purchased from R&D Systems (France). Goat anti‐mouse IgA was purchased from SouthernBio (USA). Muc2C3 was kindly provided by Prof. Dr. G. Hansson (University of Gothenburg, Sweden). Goat anti‐rabbit Alexa fluor 488 and Donkey anti‐rabbit Alexa fluor 488 was purchased from ThermoFischer (The Netherlands).

### 2.6 Histopathology

Colon sections were stained with Gram according to standard procedures of the Department of Pathology of the University Medical Center Utrecht as described previously (Hendrickx et al., 2015; Robertson, Savage, Reis‐Filho, & Isacke, 2008). The sections were analyzed by light microscopy (Nikon Eclipse E800M).

### 2.7 Scanning electron microscopy

Mouse colon tissue from day 1 was fixed in 2% glutaraldehyde after necropsy. The intact colon sections were washed twice with PBS to remove the excessive fixative. Colons were dehydrated using 5 ml of 25% and 50% ethanol‐PBS, 75% and 90% ethanol‐H_2_O and 100% ethanol. The ethanol was then substituted by 50% ethanol‐hexamethyldisilazane (HMDS) followed by 100% HMDS (Sigma, The Netherlands). The sections were air‐dried overnight, mounted onto 12 mm specimen stubs (Agar Scientific, The Netherlands) and coated with 4 nm gold using a Quorum Q150R sputter coater at 20 mA. The sections were then analyzed with a Phenom PRO Table‐top scanning electron microscope (PhenomWorld, The Netherlands).

### 2.8 Confocal laser scanning microscopy

Colon sections were prepared for immune fluorescence as described elsewhere (Hendrickx et al., 2015; Robertson et al., 2008)**.** In brief, colon sections (4 μm) on glass slides were deparaffinized with 100% xylene. The sections were rehydrated with consecutive incubations in 100% ethanol, 90% ethanol, 70% ethanol and water. Antigen was retrieved by boiling the slides (95°C) in 0.01 mol/L Na‐citrate buffer (pH = 6) for 20 min. The sections were cooled down in the same buffer and were rinsed with water. The slides were incubated for 1 hr with 200 μl of primary antibody (either anti‐Muc‐2, anti‐E‐cadherin, anti‐cadherin‐17, anti‐IgA or anti‐CD11b) all at a dilution of 1:100 in Hank's Balanced salt solution supplemented with 1% bovine serum albumin (HBSS‐BSA). After incubation, tissue was washed with 25 ml HBSS‐BSA. The slides were subsequently incubated for 1 hr with 200 μl of secondary Alexa fluor 488 conjugated antibody diluted 1:500 and TOPRO3 (LifeTechnologies) diluted 1:1000 in HBSS‐BSA in the dark. As negative controls, slides were incubated without primary antibody, but only with secondary antibody Goat anti‐rabbit Alexa fluor 488 or Donkey anti‐rabbit Alexa fluor 488. After staining, the sections were washed with 25 ml HBSS‐BSA and stained with FM5‐95 (1 μg/ml) diluted 1:1000 in H_2_O and covered with glass coverslips using Prolong Gold anti‐fade reagent (Invitrogen). Slides were analyzed using confocal laser scanning microscopy (CLSM DFC360 FX Digital Camera Kit, Leica SP5). Images were analyzed with Leica LAS AF software.

### 2.9 Quantification of IgA^+^ and CD11b^+^IgA^+^ cells

Confocal images obtained from immuno fluorescence experiments using specific anti‐mouse IgA and CD11b antibodies were used to enumerate IgA^+^ cells and CD11b^+^IgA^+^ cells from mouse colonic crypts and underlying lamina propria. Green signal in the colonic crypts and underlying lamina propria represented either IgA^+^ cells or CD11b^+^IgA^+^ cells. The number of positive IgA^+^ or CD11b^+^IgA^+^ cells per crypt was enumerated and analyzed using Graphpad Prism version 7.0.

### 2.10 Immunoglobulin A ELISA

An Enzyme‐linked immunoabsorbent assay (ELISA) was performed using a Ready‐Set‐Go mouse IgA kit (Thermofischer, The Netherlands). The ELISA plate was coated overnight using a pretitrated purified anti‐mouse IgA monoclonal antibody diluted 1:250 in PBS. The plate was washed with PBS + 0.05% Tween^20^ and blocked for 2 hr with PBS with 1% Tween^20^ 10% BSA. Subsequently, the plate was washed again and fecal extracts from the mouse intestinal colonization experiment were diluted 1:10 in PBS with 1% Tween^20^ 10% BSA, added to the appropriate wells and incubated for 2 hr. A lyophilized mouse IgA isotype (50 ng/ml) was used as a control. The plate was washed and incubated for 1 hr with a pretitrated HRP‐conjugated anti‐mouse IgA polyclonal detection antibody diluted 1:250 in PBS with 1% Tween^20^ + 10% BSA. The plate was washed again and tetramethylbenzidine was added as a substrate solution. The reaction was stopped using 2N H_2_SO_4_. The plate was read at OD of 450 nm on a plate reader (Biorad). The ELISA was performed in triplicate, the values corrected for the amount of feces obtained per mouse, and plotted.

### 2.11 Statistical analyses

All data is expressed as mean values ± SEM. The differences between groups were analyzed with an ordinary one‐way ANOVA and Tukey's multiple comparisons test using Graphpad Prism version 7.0. Values of *p *<* *.05 were considered statistically significant.

## 3 RESULTS

### 3.1 Mucus layer during early intestinal colonization by *E. faecium*

To study the primary mucosal interaction of multiantibiotic‐resistant *E. faecium* E1162 with the intestine, a mouse intestinal colonization experiment was performed. In the mouse intestinal colonization model (Figure 1a,b), antibiotic‐treated mice that were inoculated with *E. faecium* E1162 (group 1) yielded high‐density (>1 × 10^10^ CFU/gram feces) colonization on day 1, while no enterococci were detected on Slanetz‐Bartley agar in all other groups (data not shown). The effect of antibiotic treatment on the mucus layer in the mouse intestine was examined using confocal imaging. This revealed that the colonic Muc‐2 mucus layer was affected during antibiotic treatment in groups 1 and 2 (n* *=* *12), as a severe reduction in the Muc‐2 layer was observed after 6 hr of antibiotic treatment on day −2 (Figure 1c). The Muc‐2 layer remained reduced on day 0 to day 1 during the experiment, also in mice that were inoculated with E1162 on day 0 (group 1). Mice that were not treated with antibiotics (groups 3 and 4) showed no reduced mucus layer (n* *=* *12). In these animals, the colonic mucus layers were unaffected and thick in diameter. The conjugate controls used in this study did not yield any aspecific green Alexa Fluor 488 signal (Figure 1d).

### 3.2 Localization of *E. faecium* E1162 in the mouse colon

**Figure 2:**
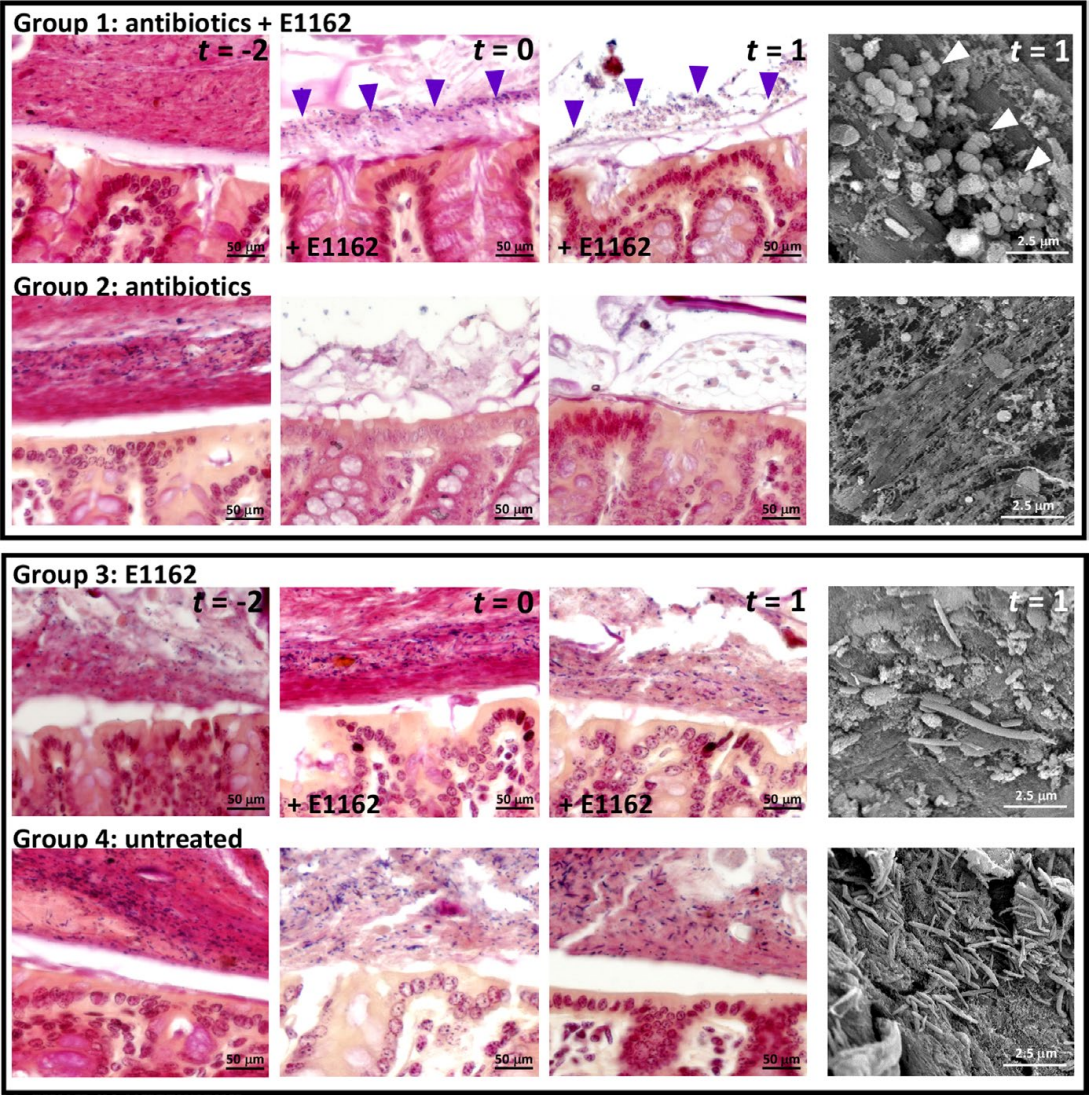
Localization of *E. faecium* in the colon during antibiotic treatment. (a) Gram‐staining on Carnoy's fixed mouse colon sections. Bacteria were visualized by Gram staining in all four mouse groups on days −2, 0, and 1. In red: Gram‐negative bacteria. In purple: Gram‐positive bacteria. Arrows indicate Gram‐positive cocci. Right panels: scanning electron microscopy on glutaraldehyde‐fixed and dehydrated colon sections from day 1. Arrows indicate diplococci

To determine the location of *E. faecium* in the colon, Gram‐staining and scanning electron microscopy (SEM) were performed on Carnoy's fixed and glutaraldehyde‐fixed mouse colon tissue respectively. In all mice, Gram‐positive and Gram‐negative microbiota was detected in the intestine as purple and pink bacteria on day ‐2 (Figure 2). However, on day 0, the microbiota was not visible in antibiotic treated animals of group 2, which remained to day 1 (n* *=* *4). Only in animals of group 1, that were treated with antibiotics and inoculated with *E. faecium* E1162, purple Gram‐positive cocci were observed at the apical side of the epithelium 6 hr after inoculation at day 0, and these cocci remained there during the course of the experiment from day 0 to day 1 (n* *=* *4). SEM on mouse colons confirmed the Gram‐staining on day 1. Diplococci were detected in animals of group 1 in which a high density of *E. faecium* E1162 was detected in the feces (see above). In animals of group 2, bacteria were rarely observed, while in animals not treated with antibiotics a diverse microbiota was observed (group 3 and 4; Figure 2). Thus, while the Muc‐2 layer is reduced after 6 hr of antibiotic treatment on day −2, *E. faecium* E1162 is the dominant bacterium at the apical side of the intestinal epithelium within 6 hr on day 0.

### 3.3 E‐cadherin and cadherin‐17 junctions during *E. faecium* outgrowth

**Figure 3:**
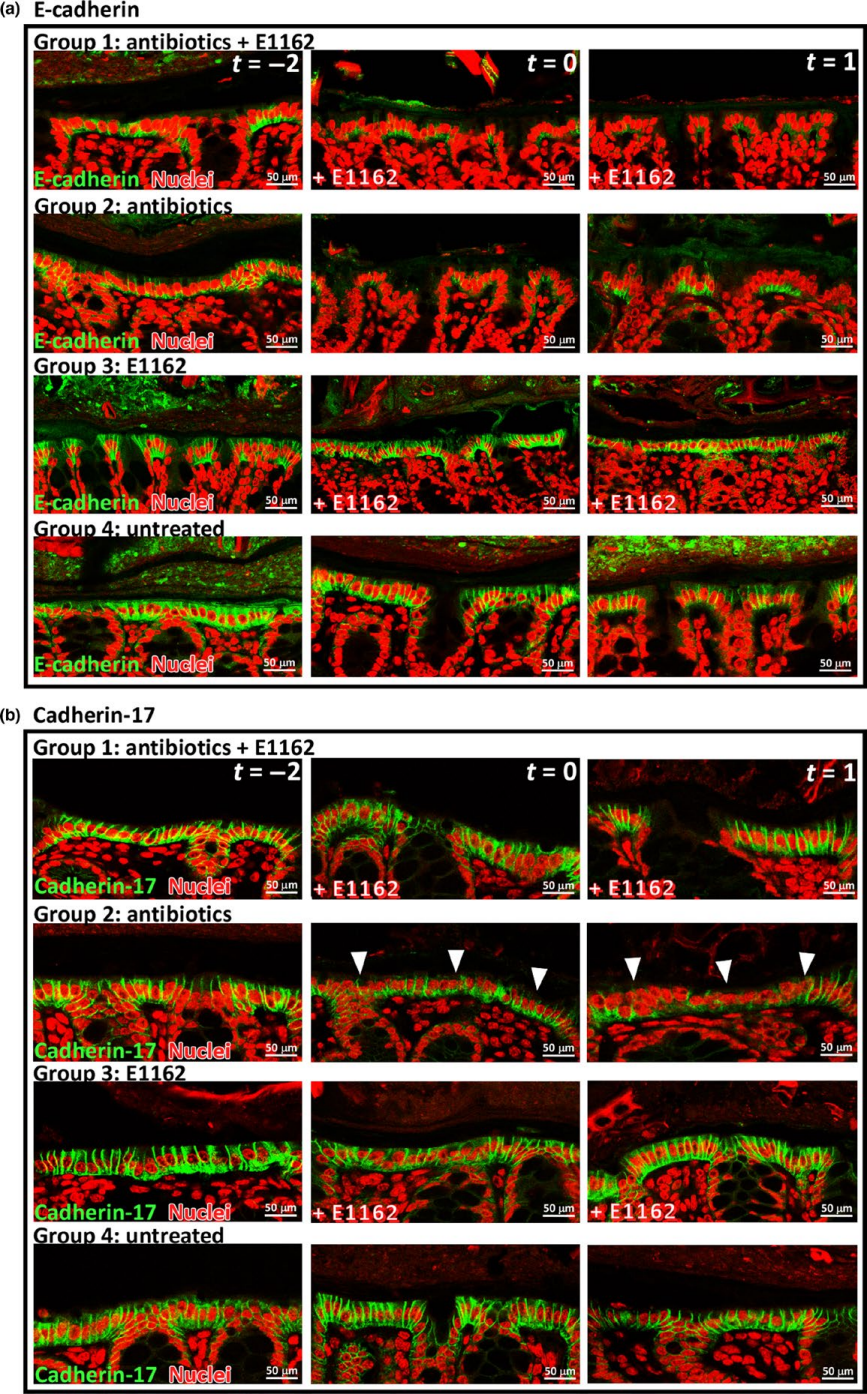
E‐cadherin and cadherin‐17 junctions are affected in the mouse colon during antibiotic treatment. (a) In immunofluorescence experiments, E‐cadherin was stained in all four mouse groups on days −2, 0, and 1. In green: rabbit anti‐mouse E‐cadherin + goat anti‐rabbit Alexa Fluor 488. In red: TO‐PRO‐3 nucleic acid stain. (b) Cadherin‐17 was stained in all four mouse groups on days −2, 0, and 1. In green: rabbit anti‐mouse cadherin‐17+ goat anti‐rabbit Alexa Fluor 488. For both (a) and (b), in red: TO‐PRO‐3 nucleic acid stain. The images are representative of two animals per group. Arrows indicated affected cadherin‐17 junctions

To analyze the effect of antibiotics and *E. faecium* E1162 colonization on E‐cadherin and cadherin‐17 junctions, confocal imaging was performed on mouse colon sections. This showed that on day −2, day 0, and day 1, the E‐cadherin junctions were highly affected in antibiotic treated mice of groups 1 and 2 (n* *=* *12; Figure 3a). The junctions were shorter and damaged within 6 hr after the start of antibiotic treatment, which correlates in time with the reduction in the Muc‐2 layer (Figure 1c). Animals not treated with antibiotics (groups 3 and 4) revealed normal adherens junctions (n* *=* *12). Analysis of cadherin‐17 junctions revealed that in mice of group 2 that received only antibiotics, cadherin‐17 junctions were shorter on day 0 and day 1 (n* *=* *4) (Figure 3b). However, in group 1 mice that were treated with antibiotics and inoculated with *E. faecium* E1162, the cadherin‐17 junctions remain unaffected on days −2 to 1. In mice not treated with antibiotics (groups 3 and 4) the cadherin‐17 junctions were also unaffected (n* *=* *12) (Figure 3b). Thus, while the E‐cadherin adherens junctions are affected during antibiotic treatment and *E. faecium* colonization, the cadherin‐17 junctions were not.

### 3.4 Increased IgA^+^ and CD11b^+^IgA^+^ cells during antibiotic‐driven *E. faecium* colonization

**Figure 4:**
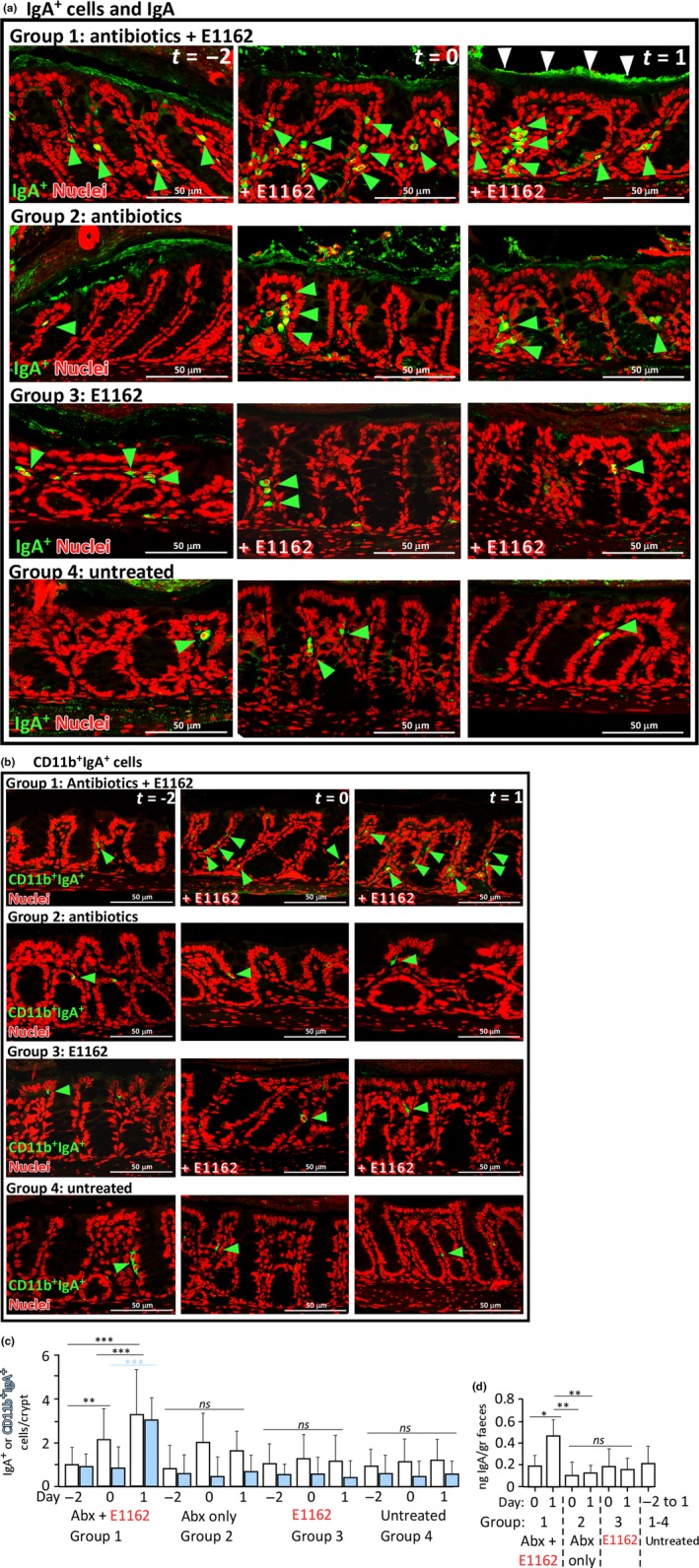
IgA^+^ and CD11b^+^IgA^+^ plasma cells and the IgA response to *E. faecium* in the colon. (a) In immuno fluorescence experiments, IgA^+^ plasma cells were stained in all four mouse groups on days −2, 0, and 1. Green arrows indicate IgA^+^ plasma cells. White arrows indicate agglutinated IgA. In green: goat anti‐mouse IgA + donkey anti‐goat Alexa Fluor 488. (b) CD11b^+^IgA^+^ plasma cells were stained, similar as in (A). Green arrows indicate CD11b^+^IgA^+^ plasma cells. For both (a) and (b), in red: TO‐PRO‐3 nucleic acid stain. The images are representative of two animals per group. (C) Quantification of IgA^+^ plasma cells (white bars) and CD11b^+^IgA^+^ plasma cells (blue bars) per crypt from colon sections from all mice (n* *=* *24). (d) Quantification of IgA by a specific ELISA from fecal extracts of mice from the groups of mice in this experiment, similar as in (a). IgA concentration was corrected for the amount of feces obtained per mouse to yield ng IgA per gram mouse feces. The ELISA was repeated three times, data averaged, and plotted. IgA concentrations for mice from groups 1 to 4 from day ‐2 was averaged and termed “untreated”, since mouse feces was obtained on each sampling day at 10 am (See Figure 1b). For (C) and (D): *ns*, not significant; *P < *.05 is regarded significant

After analysis of the initial effects of cephalosporin‐mediated *E. faecium* colonization on the Muc‐2 layer, E‐cadherin and cadherin‐17 junctions, we next analyzed IgA secretion by IgA^+^ and CD11b^+^IgA^+^ plasma cells in the colons of these mice. In antibiotic‐treated animals that received *E. faecium* E1162 (group 1), the IgA^+^ plasma cells within the lamina propria were increased on day 0 and day 1 (Figure 4a). Also CD11b^+^IgA^+^ cells were most abundant in mice of group 1 (Figure 4b). At day 1, IgA is present as a layer at the apical side of the epithelium and this was most pronounced in mice of group 1 (n* *=* *4), while this was less prominent in mice of group 2 (n* *=* *4) and absent in mice of the other two groups (Figure 4a). Next, the number of either IgA^+^ or CD11b^+^IgA^+^ plasma cells was enumerated per crypt among the mouse groups (Figure 4c). This revealed that only in group 1 the number of IgA^+^ cells was significantly increased from 0.98 on day ‐2 to 1.94 on day 0 and 3.16 on day 1 (*p *<* *.05). No significant differences were observed in the other three groups. In line with this, also the number of CD11b^+^IgA^+^ cells per crypt increased significantly only in group 1; from 0.96 on day −2 and 0.94 on day 0 to 3.17 on day 1 (*p *<* *.05). Compared to mice not treated with antibiotics (groups 3 and 4) the number of IgA^+^ and CD11b^+^IgA^+^ plasma cells doubled in group 1 (Figure 4c). Thus, during antibiotic treatment the host has already 6 hr after *E. faecium* colonization an increased level of IgA^+^ and CD11b^+^IgA^+^ cells.

### 3.5 Mucosal IgA response to *E. faecium* E1162 colonization

The increased number of CD11b^+^IgA^+^ cells and the clearly visible IgA lining at the apical side of the epithelium suggest an increased IgA production in the mouse colon upon antibiotic treatment and exposure to *E. faecium* E1162. To further validate this an ELISA was performed to measure IgA concentration in fecal extracts of the mice (Figure 4d). The IgA concentration in group 1 significantly increased from 0.39 ng IgA/gram feces on day 0 to 0.56 ng IgA/gram feces on day 1, while in groups 2 and 3 on day 0 and 1 the IgA concentration did not change significantly and ranged between 0.21 and 0.35 ng IgA/gram feces. This was similar for animals that were untreated (group 4), including animals from groups 1 to 3, in which IgA was measured in feces at day −2 before receiving antibiotics or *E. faecium* E1162. Taken together, the data presented here suggests an elevated IgA response by IgA^+^ and CD11b^+^IgA^+^ plasma cells in antibiotic treated animals that are exposed to *E. faecium* E1162.

## 4 DISCUSSION

The mammalian intestines are densely populated by microbiota, which has an essential role in metabolism and colonization resistance to invading pathogens. However, antibiotic treatment is known to dramatically alter the microbiota leading to a reduction in colonization resistance, which allows the proliferation of drug‐resistant bacteria in the gut, such as *Clostridium dificile*,* Enterococcus faecalis*, as well as *E. faecium* (Britton & Young, 2014; Hendrickx et al., 2015; Rigottier‐Gois et al., 2015; Steck et al., 2011; van der Heijden et al., 2014). In this study, we focused on the initial stage of gastrointestinal colonization by *E. faecium* E1162 during antibiotic treatment. In our mouse intestinal colonization model for outgrowth of a hospital‐associated pathogen employed here, cephalosporin antibiotic treatment affected the distribution of intestinal bacteria after one day, and also significantly reduced the colonic Muc‐2 mucus layer. It has been shown previously that antibiotics can mediate the reduction in the Muc‐2 layer (Hendrickx et al., 2015; Wlodarska et al., 2011), likely by creating intestinal dysbiosis, but here we show that this can occur already within 6 hr after administration of the first ceftriaxone injection. During antibiotic treatment and after inoculation, multiresistant *E. faecium* E1162 colonized at the apical side of the colonic epithelium. Previous work showed that *E. faecium* E1162 agglutinates in the gut of mice by E‐cadherin, polymeric immunoglobulin receptor and IgA at the apical side of the epithelium (Hendrickx et al., 2015). In the current study, we observed a similar agglutination of *E. faecium* already 6 hr after inoculation. We speculate that this may prevent translocation from the gut into the bloodstream and subsequent dissemination within the host.

The colonic epithelial cell layer, partially maintained by E‐cadherin and cadherin‐17 junctions, forms a barrier against possible pathogenic bacteria. While the E‐cadherin and cadherin‐17 junctions were altered during antibiotic treatment within 6 hr and remained damaged until the end of the experiment at day 1, the cadherin‐17 junctions were unaffected in antibiotic‐treated mice during *E. faecium* colonization or in untreated animals. This suggests that *E. faecium* E1162 colonization may positively influence the occurrence of cadherin‐17‐based junctions between intestinal epithelial cells. Cadherin‐17 is distributed along the lateral cell membrane, but is not present in E‐cadherin junctions. Previous studies have speculated that due to the high lateral mobility of cadherin‐17, this cell adhesion molecule could be responsible for fast and flexible initial formation of adhesive contacts between cells during development and tissue regeneration (Bartolmäs, Hirschfeld‐Ihlow, Jonas, Schaefer, & Geßner, 2012; Baumgartner, 2013; Wendeler et al., 2007). We speculate that while the E‐cadherin and cadherin‐17 junctions are affected during antibiotic treatment, cadherin‐17 may be dispensable for this loss in the presence of *E. faecium*. Whether this phenomenon is specific for *E. faecium* and whether *E. faecium* triggers cadherin‐17 expression and regeneration in our model remains to be investigated.

Upon intestinal colonization with *E. faecium* during antibiotic treatment, numbers of IgA^+^ and CD11b^+^IgA^+^ plasma cells increased, which is suggestive of an early host response against this hospital‐associated pathogen. Intestinal CD11b^+^IgA^+^ plasma cells, which are a subpopulation of IgA^+^ plasma cells, require microbial stimulation for high‐level IgA production (Kunisawa et al., 2013). We are the first to show that during cephalosporin antibiotic treatment in our intestinal colonization model, the population of IgA^+^ plasma cells and CD11b^+^IgA^+^ plasma cells increased in the colon and produced elevated levels of IgA in response to *E. faecium* colonization. The number of CD11b^+^IgA^+^ cells did not increase in animals that were treated with antibiotics only, confirming that this subpopulation of IgA^+^ plasma cells require microbial stimulation (Kunisawa et al., 2013). IgA agglutinates at the apical side of the colonic epithelial cells to maintain a spatial segregation, and likely to exclude *E. faecium* from the host.

In summary, we analyzed important components of the protective barrier (Muc‐2, E‐cadherin, cadherin‐17, IgA) during the primary stage of antibiotic‐mediated colonic colonization of *E. faecium* in mice. Understanding gastrointestinal colonization by *E. faecium* at the molecular level may provide new insights leading to future treatments that reduce high‐density colonization with this drug‐resistant microbe in antibiotic treated hospitalized patients. This knowledge may also help to prevent outgrowth and subsequent infections and transmission of other opportunistic antibiotic‐resistant pathogens.

## CONFLICT OF INTEREST

None declared.
